# Vaccine-Induced Lichen Planus in a 12-Year-Old Patient: A Rare Pediatric Case

**DOI:** 10.7759/cureus.84521

**Published:** 2025-05-21

**Authors:** Sara Nejjari, Inas Chikhaoui, Ghita Basri, Soumiya Chiheb

**Affiliations:** 1 Dermatology, Cheikh Khalifa International University Hospital, Mohammed VI University of Health Sciences, Casablanca, MAR

**Keywords:** dermoscopy findings, drug-induced lichen planus, inflammatory skin disorder, pediatric case, tetanus vaccine

## Abstract

We report a rare case of vaccine-induced lichen planus (LP) in a 12-year-old male patient who developed generalized pruritic, violaceous, flat-topped papules and plaques two weeks after receiving a tetanus vaccine following a puncture wound. Clinical evaluation, dermoscopic examination showing Wickham striae and follicular hyperkeratosis, and histopathology confirmed the diagnosis. Vaccine-induced LP remains an uncommon but possible immune-mediated adverse reaction. Dermoscopy proved valuable for non-invasive diagnosis, aiding in distinguishing LP from other dermatologic conditions. This case emphasizes the importance of considering vaccine-induced LP in pediatric patients presenting with new-onset lesions post-vaccination and highlights the role of dermoscopy in early recognition and management.

## Introduction

Lichen planus (LP) is a chronic, immune-mediated inflammatory skin disorder characterized by pruritic, violaceous, flat-topped papules. It is commonly associated with triggers such as medications, viral infections, and mechanical trauma [1,2]. Although drug- and infection-induced LP are well recognized, vaccine-induced LP remains rare, with few cases reported in the literature [3]. Vaccines may trigger LP through immune activation, potentially leading to a loss of tolerance to epidermal antigens [4]. We report a rare pediatric case of generalized LP occurring two weeks after a tetanus vaccination, highlighting the importance of considering this etiology in the differential diagnosis of new-onset lichenoid eruptions.

## Case presentation

A 12-year-old male patient, who was up to date on routine childhood immunizations and had no prior adverse reactions to vaccines and no personal or familial history of autoimmune or dermatological disorders, presented to the dermatology clinic with a two-week history of intensely pruritic skin lesions. The onset occurred shortly after receiving a tetanus toxoid vaccine (Tdap, combined with diphtheria and pertussis) administered as part of post-exposure prophylaxis following a puncture wound from rusty metal.

Cutaneous examination

**Figure 1:**
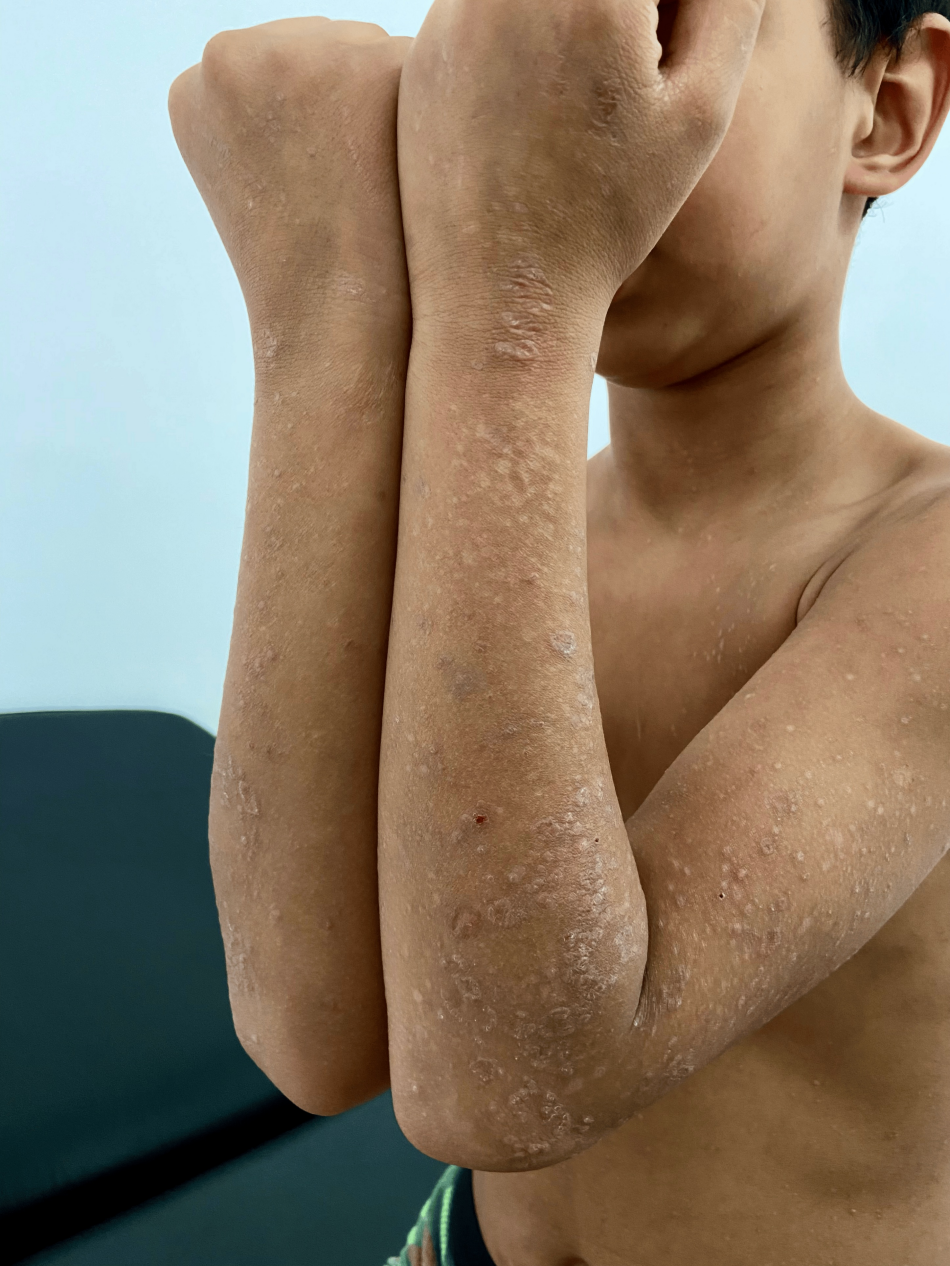
Lichen planus on the arms and forearms characterized by flat-topped, violaceous papules with a fine, white lacy pattern

**Figure 2:**
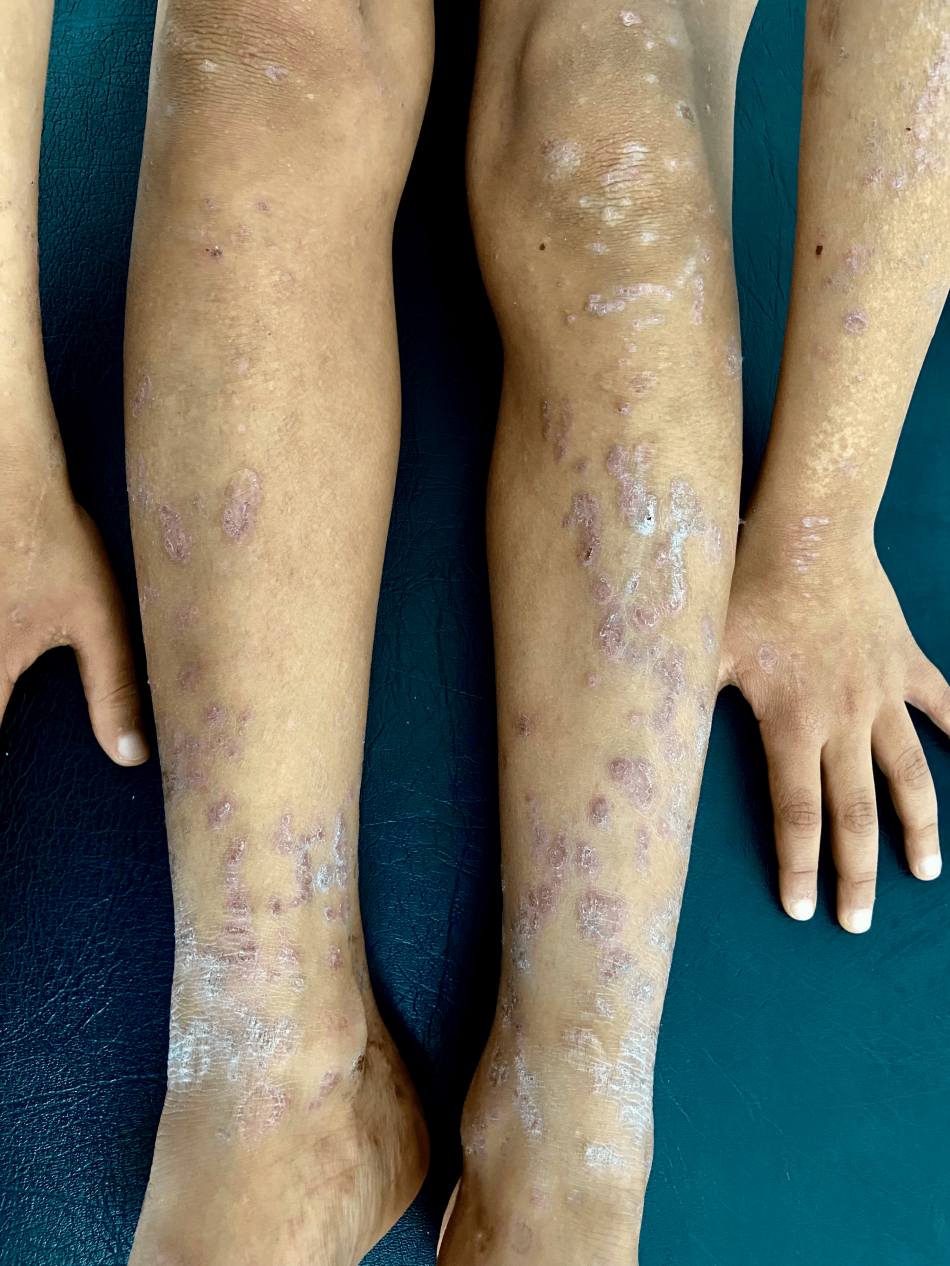
Lichen planus on the lower limbs shown by violaceous papules with a white lacy pattern

**Figure 3:**
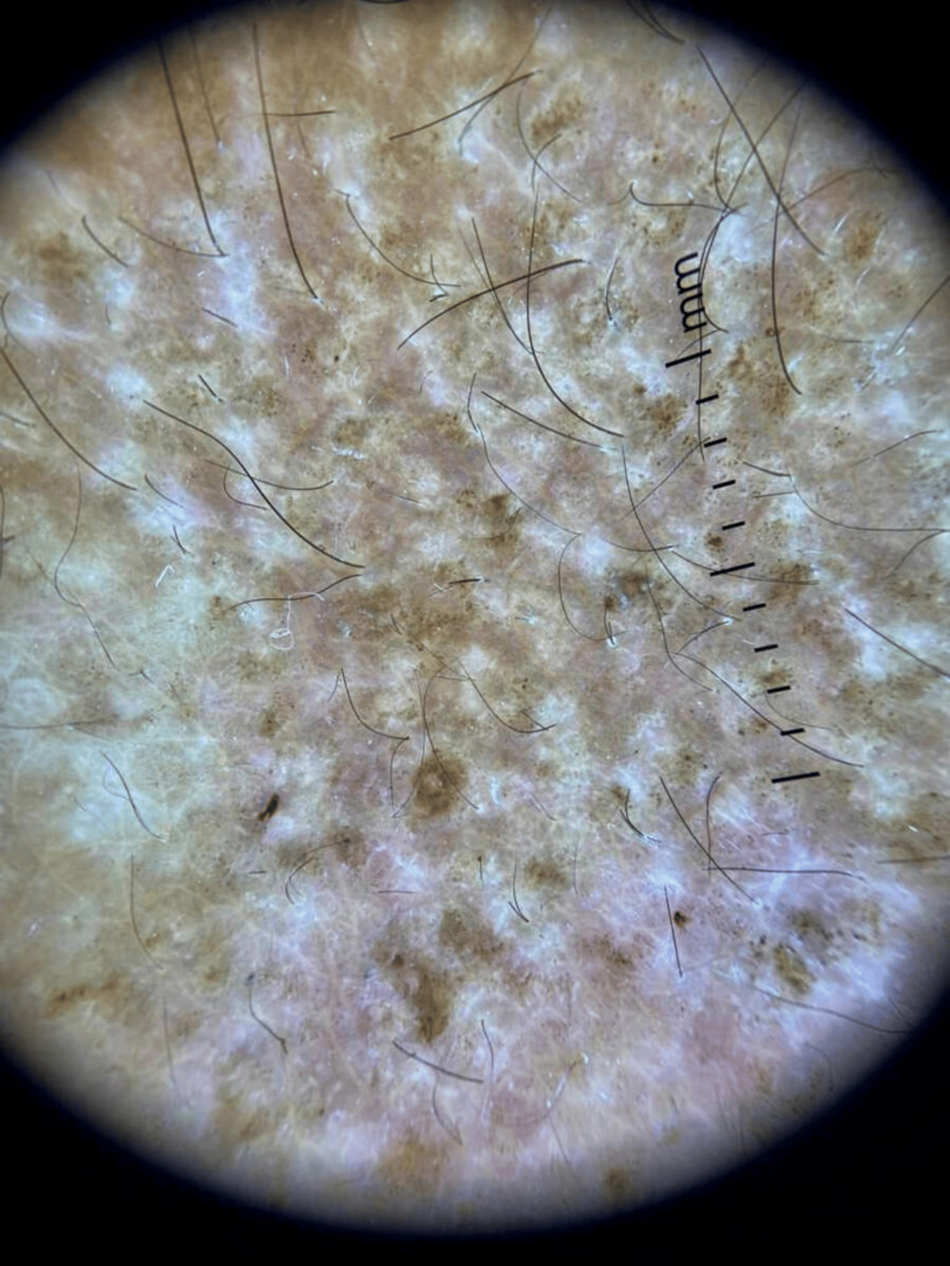
Dermoscopy of lichen planus showing flat-topped, violaceous papules with Wickham striae

On physical examination, the patient had multiple violaceous, polygonal, flat-topped papules and plaques symmetrically distributed over the trunk, forearms (Figure 1), dorsal hands, thighs, and legs (Figure 2). The lesions were discrete in some areas and confluent in others, particularly on the lower back. Several papules demonstrated fine, white reticulated lines, Wickham striae, especially under dermoscopic evaluation using a polarized light dermatoscope (Figure 3). The skin surface appeared dry, with mild follicular hyperkeratosis noted on the limbs. 

Mucosal examination

An intraoral examination revealed no mucosal involvement; the buccal mucosa, tongue, and gingiva were intact and free from any erosions, white striae, or pigmented lesions. Similarly, the genital mucosa showed no abnormalities. Nail and scalp examinations were unremarkable, with no signs of nail ridging, pterygium formation, or scalp scaling.

Histopathological confirmation

**Figure 4:**
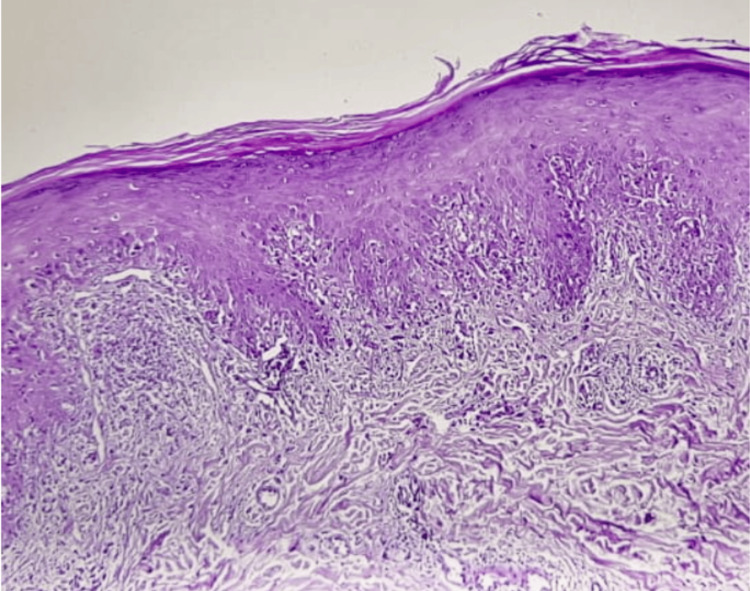
Histological section of the lesional skin (H&E stain, ×40) showing a thin layer of orthokeratosis keratin, dermal inflammatory infiltrate in bands, and lymphocytic infiltrate penetrating the basal layers H&E: hematoxylin and eosin

While dermoscopy aids in non-invasive diagnosis, histopathological confirmation remains the gold standard for diagnosing LP. A 4-mm punch biopsy taken from a lesion on the left forearm confirmed the diagnosis of LP. Histological analysis showed hypergranulosis, saw-tooth acanthosis of the epidermis, a band-like lymphocytic infiltrate at the dermoepidermal junction, and apoptotic keratinocytes (Civatte bodies), findings consistent with LP (Figure 4).

Management and treatment

The patient was started on a topical high-potency corticosteroid (clobetasol propionate 0.05% cream) applied twice daily to the affected areas for three weeks, along with a moisturizing emollient to reduce xerosis. Due to the severity of pruritus, a non-sedating oral antihistamine (cetirizine 10 mg daily) was also prescribed. The patient and parents were counseled on the benign, self-limiting nature of the disease and instructed to avoid scratching to prevent Koebnerization. Systemic therapy was not initiated as the disease was localized and moderately severe and responded well to topical treatment alone.

Follow-up and outcome

At the four-week follow-up, the patient showed marked clinical improvement. The papules had flattened, pruritus was significantly reduced, and post-inflammatory hyperpigmentation was noted in previously active sites. No new lesions appeared during treatment, and no systemic therapy was required. Continued use of emollients and tapering of the topical corticosteroid over the following weeks were advised. At the two-month mark, the disease was considered in remission.

## Discussion

Vaccine-induced LP is a rare but increasingly recognized phenomenon, with reported cases following various immunizations, including hepatitis B, influenza, human papillomavirus (HPV), and, most recently, COVID-19 vaccines [3,4]. The precise pathogenesis remains incompletely understood, but it is generally believed to involve a dysregulated immune response. In predisposed individuals, antigen presentation following vaccination may activate cytotoxic T lymphocytes and lead to a cascade of cytokine release, ultimately resulting in an autoimmune attack on basal keratinocytes [5]. This immunologic mechanism mirrors the pathophysiology of idiopathic LP, further supporting the plausibility of a vaccine-related trigger.

In the present case, the close temporal association, approximately two weeks, between the administration of a tetanus vaccine and the onset of clinical symptoms strongly suggests a vaccine-induced etiology. This inference is reinforced by the absence of other identifiable precipitating factors such as infections, medications, or systemic diseases. Moreover, the patient exhibited classic clinical, dermoscopic, and histopathological features of LP, fulfilling established diagnostic criteria and supporting the diagnosis.

Dermoscopy played a critical role in this case by facilitating early, non-invasive identification of LP-specific patterns, including Wickham striae and follicular hyperkeratosis. These findings are well-documented hallmarks of LP and have been shown to improve diagnostic accuracy, particularly in atypical or early-stage presentations. In pediatric patients, where performing skin biopsies can be challenging due to procedural anxiety and parental concerns, dermoscopy offers a valuable alternative. It allows for confident diagnosis and management decisions while minimizing the need for invasive procedures.

The rarity of LP in the pediatric population further underscores the significance of this case. While LP is more frequently encountered in adults, its occurrence in children remains relatively uncommon, and vaccine-induced forms are even rarer. This makes each reported case a valuable addition to the medical literature, contributing to a broader understanding of the potential spectrum of adverse vaccine reactions.

Clinicians should maintain a high index of suspicion for vaccine-related cutaneous eruptions, particularly when there is a clear temporal link and no other plausible etiology. Prompt recognition allows for appropriate treatment, typically involving topical corticosteroids and antihistamines, which were effective in this case. The favorable clinical outcome, characterized by symptom resolution and absence of recurrence, is consistent with previous reports indicating a generally benign and self-limiting course in vaccine-induced LP [6,7].

As global immunization efforts continue, it is essential to balance vaccine safety monitoring with the overall benefits of vaccination. While rare, adverse events such as LP should be systematically documented and investigated to enhance post-marketing surveillance and guide evidence-based clinical practice. Raising awareness among healthcare providers is crucial to ensuring early identification, appropriate counseling of patients and caregivers, and optimal therapeutic outcomes.

## Conclusions

This case highlights the importance of considering vaccine-induced LP in the differential diagnosis of new-onset LP, particularly in pediatric patients where a clear temporal relationship exists between vaccination and the emergence of cutaneous symptoms. LP is relatively uncommon in the pediatric population, making such presentations particularly noteworthy and deserving of clinical attention. Early recognition of this potential adverse event is crucial, not only to prevent misdiagnosis and inappropriate treatment but also to contribute to a more comprehensive understanding of the spectrum of vaccine-related cutaneous reactions.

Dermoscopy plays a pivotal role as a non-invasive diagnostic tool, offering characteristic features that can aid clinicians in distinguishing LP from other dermatoses, thus expediting accurate diagnosis and management. As global vaccination programs continue to expand, especially in response to emerging infectious threats, healthcare providers should remain vigilant for rare but significant adverse cutaneous events. Awareness and documentation of such cases can enhance post-marketing surveillance efforts, inform future vaccine safety assessments, and ultimately guide clinical practice in managing vaccine-related dermatologic conditions in children.
